# A brief diet intervention can reduce symptoms of depression in young adults – A randomised controlled trial

**DOI:** 10.1371/journal.pone.0222768

**Published:** 2019-10-09

**Authors:** Heather M. Francis, Richard J. Stevenson, Jaime R. Chambers, Dolly Gupta, Brooklyn Newey, Chai K. Lim

**Affiliations:** 1 Psychology Department, Macquarie University, Sydney, NSW, Australia; 2 Sydney Integrative Medicine, Level 1, Sydney, NSW, Australia; 3 Cooper St Clinic, Sydney, NSW, Australia; 4 Biomedical Sciences, Macquarie University, Sydney, NSW, Australia; National Cancer Center, JAPAN

## Abstract

There is strong epidemiological evidence that poor diet is associated with depression. The reverse has also been shown, namely that eating a healthy diet rich in fruit, vegetables, fish and lean meat, is associated with reduced risk of depression. To date, only one randomised controlled trial (RCT) has been conducted with elevated depression symptoms being an inclusion criterion, with results showing that a diet intervention can reduce clinical levels of depression. No such RCTs have been performed in young adults. Young adults with elevated levels of depression symptoms and who habitually consume a poor diet were randomly allocated to a brief 3-week diet intervention (Diet Group) or a habitual diet control group (Control Group). The primary and secondary outcome measures assessed at baseline and after the intervention included symptoms of depression (Centre for Epidemiological Studies Depression Scale; CESD-R; and Depression Anxiety and Stress Scale– 21 depression subscale; DASS-21-D), current mood (Profile of Mood States), self-efficacy (New General Self-Efficacy Scale) and memory (Hopkins Verbal Learning Test). Diet compliance was measured via self-report questionnaires and spectrophotometry. One-hundred-and-one individuals were enrolled in the study and randomly assigned to the Diet Group or the Control Group. Upon completion of the study, there was complete data for 38 individuals in each group. There was good compliance with the diet intervention recommendations assessed using self-report and spectrophotometry. The Diet group had significantly lower self-reported depression symptoms than the Control Group on the CESD-R (*p* = 0.007, Cohen’s *d* = 0.65) and DASS-21 depression subscale (*p* = 0.002, Cohen’s *d* = 0.75) controlling for baseline scores on these scales. Reduced DASS-21 depression subscale scores were maintained on follow up phone call 3 months later (*p* = .009). These results are the first to show that young adults with elevated depression symptoms can engage in and adhere to a diet intervention, and that this can reduce symptoms of depression. The findings provide justification for future research into the duration of these benefits, the impacts of varying diet composition, and their biological basis.

## Introduction

There has been a global shift from a healthy diet pattern high in complex carbohydrate and fibre, to a diet high in processed foods, saturated fats and refined sugars [1] (hereafter HFS). The literature now strongly suggests that poor diet quality is associated with an increased risk of depression [2]. Diet is therefore a modifiable risk factor for depression, which would be a good target for early intervention. However, while there is convincing observational evidence for a link between diet quality and depression, the evidence for a causal relationship is still emerging, particularly in relation to young adults.

Several systematic reviews and meta-analyses show a relationship between diet quality and depression [3–6]. A meta-analysis showed that healthy diet regardless of pattern (e.g. Mediterranean, vegetarian, Tuscan) was linearly associated with reduced incidence of depression [5]. However, In two meta-analyses there was no significant relationship between Western-style diet or unhealthy diet patterns as being associated with increased odds of depression, however there were fewer studies available for analyses [4, 5]. In a systematic review of studies involving children and adolescents (aged 4.5–18 years), unhealthy diet patterns were associated with poorer mental health outcomes and there was a trend for the relationship between healthy diet quality and better mental health [7]. However, the findings are attenuated by the fact that diet was not associated with depression incidence in studies that used a clinical depression diagnosis as an outcome measure, or those that controlled for baseline depression severity [5], and no association was found with psychiatrist diagnosed depression [8]. Randomised controlled trials are therefore required in order to establish that the relationship observed in these studies is causal.

A meta-analysis of 16 randomised controlled trials (RCTs) that involved some type of diet-improvement showed that diet interventions overall reduced depressive symptoms [9]. However, in most of these, depression symptoms were a secondary outcome of interest, and many studies have compared the effect of two differing diets, or involved lifestyle change such as diet, exercise and sleep combined (see [10] for a review). To date, there has only been one randomised controlled trial examining a diet intervention for individuals with a clinical diagnosis of depression. The SMILES trial showed that in adults with a mean age of 40 years, a 12-week healthy diet intervention improved ratings of depression on a clinical rating scale compared to a social support control group [11]. To our knowledge, only one study has examined the effect of a diet intervention in young adults (females aged 18–30), however, depressive symptoms were not measured [12]. Rather, current mood was assessed using the Profile of Mood States, with no significant difference found between the ‘diet change’ group compared to the ‘no change’ control group. This may be due to the fact that the Profile of Mood States is a measure of current, transient mood, and in relation to depressed mood, only asks whether the respondent currently feels depressed, downhearted, miserable and unhappy. This is opposed to questionnaires assessing depression symptoms over a longer duration, or clinical diagnostic interviews.

Adolescence and young adulthood are a period where there is increased risk of depression, and these are also critical periods for establishing health patterns—such as diet–which will carry over into adulthood [13]. Therefore, diet improvement during this period have the potential to reduce risk of depression and confer other physical health benefits throughout the lifespan. Thus, the overall aims of the current study were i) to investigate whether young adults with elevated depression symptoms would comply with a brief, 3-week diet intervention, ii) whether this can improve symptoms of depression; and iii) whether compliance to the diet would be associated with improvement in depression symptoms. We hypothesised that engaging in a brief diet intervention which emphasized increasing healthy and decreasing unhealthy food intake would reduce levels of depression symptoms compared to a habitual diet control group. We further hypothesised that self-reported diet and spectrophotometry as an objective measure of diet compliance with the recommendation to increase fruit and vegetable intake [14], would improve for the diet change group over the course of the intervention, and this change would be associated with any improvements in mood. As no comparable studies have been performed previously, the data regarding maintenance of the diet recommendations and depression symptoms at 3-month follow up was exploratory. The overall aims of the study were achieved.

## Methods

### Study design

The present study was a 3-week, parallel group, single blind RCT of an intervention to improve depressed mood. The trial was registered with the Australia and New Zealand Clinical Trials Register (ACTRN12617000423314) prior to commencing recruitment. The study was conducted according to the guidelines laid down by the Declaration of Helsinki, with written informed consent obtained for each participant. The ethical framework that governs human research in Australia, The National Statement, Chapter 4.2 (Children and Young People), specifies that in terms of obtaining consent for young people, there is no specific age but rather should be judged based on cognitive capacity to consent. As all participants in the study were enrolled in tertiary education, they were treated as adults who can consent for themselves. It is on this basis that the protocol was approved by the Human Research Ethics Committee at Macquarie University (approval 520170067) and available on request from the corresponding author. Due to the impact on recruitment, serum blood samples were not collected as planned in the protocol. Participants were recruited from an undergraduate psychology course and participated for course credit, or via advertisement on the university campus and surrounds and participated for cash reimbursement. Recruitment was paused four weeks prior to exam periods to avoid the intervention overlapping with exam-related stress. Participants were randomized into either a diet change group, or habitual diet control group, based on an Excel-generated randomization schedule. Participants in both groups completed assessments of the primary and secondary measures at baseline, returning after 3 weeks (Day 21) to repeat this testing and receiving a phone call after 3 months to answer questions pertaining to diet and depression. Reporting of findings pertaining to primary and secondary outcomes was done in accordance with the APA Journal Article Reporting Standards [15].

### Participants

#### Inclusion criteria

Participants were eligible if they were aged 17–35, had a score ≥7 on the Depression, Anxiety and Stress Scale-21 Depression subscale (DASS-21-D), which corresponds with moderate or higher depression symptoms [16], and a score > 57 on the Dietary Fat and Sugar Screener (DFS), with scores >57 suggesting a poor diet that does not comply with the Australian Guide to Healthy Eating [17]. If receiving antidepressant medication or psychological therapy, participants were required to be on the same treatment for at least 2 weeks before study participation.

#### Exclusion criteria

Participants were ineligible if they were pregnant women, currently dieting, had a history of eating disorders or metabolic disease(s), history of psychological illness other than depression or anxiety, medical condition that could be adversely affected by diet change, poor proficiency in English, recent illicit drug use, or sickness in the past week.

### Intervention

Participants in the Diet Change group received the diet intervention instructions from our registered dietician via a 13-minute video, available for re-watching online as needed. The diet was developed by an Accredited Practising Dietician and was based on the Australian Guide to Healthy Eating (2003) [18], with additional recommendations to increase concordance with Mediterranean-style diets known to be associated with reduced risk of depression (2) and diet components (e.g. omega-3 fatty acids, cinnamon, turmeric) that have beneficial effects on neurological function (e.g. see [19] for a review). Participants were instructed to increase intake of vegetables (5 servings per day), fruits (2–3 per day), wholegrain cereals (3 per day), protein (lean meat, poultry, eggs, tofu, legumes; 3 per day), unsweetened dairy (3 per day), fish (3 per week), nuts and seeds (3 tablespoons per day), olive oil (2 tablespoons per day), spices (turmeric and cinnamon; 1 teaspoon most days). Conversely, they were instructed to decrease refined carbohydrate, sugar, fatty or processed meats and soft-drinks. Participants were provided a sample meal plan and recipes, a handout answering frequently asked questions and troubleshooting solutions. Given the population, focus was given to potential problems such as cost-saving and limited time for food preparation. To assist in complying with diet recommendations, participants in the diet change group each received a small hamper of food items including olive oil (Cobram Estate), natural nut butter (Mayvers), nuts and seeds (walnuts, almonds, pepitas, sunflower seeds) and spices (cinnamon, turmeric). They were told to keep their shopping receipts in order to receive a $60 gift card as reimbursement for study foods. Participants in the diet change group also received a brief, 5-minute phone call on Day 7, Day 14 to ask if they were having any difficulties adhering to the diet and troubleshoot problems with reference to the prescribed handouts. Participants in the habitual diet group were given no instructions regarding diet, and were simply asked to return after 3 weeks for follow up.

### Assessments and outcomes

Participants completed primary and secondary outcome measures, diet compliance checks and clinical data at Macquarie University for both baseline and Day 21. We chose a period of 3-weeks for the intervention because it approximates the 2–4 week time course of inflammation reduction following antidepressant treatment (see [20] for a review) and was brief enough to maximise attendance rate for post-intervention assessment. Thus, we wanted it to be long enough for the hypothesized physiological mechanisms to take effect, but not so long as to discourage attendance. Three months following the intervention, the Diet Change group participants were contacted by phone and asked ten questions to assess their diet intake and the seven items of the Depression Anxiety and Stress—depression subscale (secondary outcome), which was selected rather than the primary outcome, the Center for Epidemiological Studies Depression scale–Revised, for brevity and ease of response options when administered via telephone.

#### Primary outcome

The Centre for Epidemiological Studies Depression scale-Revised (CESD-R) was depressive symptoms post-treatment, controlling for baseline scores. The CESD-R measures symptoms defined by the American Psychiatric Association' Diagnostic and Statistical Manual (DSM-V) for a major depressive episode and has been shown to have good reliability and validity [21, 22], and has been used extensively in epidemiologic studies [23]. The CESD-R comprises 20 items asking participants to rate how often they have experienced depression symptoms “over the past week or so” with scores ranging from 0 = “Not at all or less than one day” to 3 = “Nearly every day for 2 weeks”. Scores range from 0 to 60, with higher scores representing greater symptom severity.

#### Secondary outcomes

Depression symptoms were additionally assessed using the Depression Anxiety Stress Scale-21 (DASS-21) [16]. Participants were asked to rate how often over the past week they had experienced depression symptoms on a 4-point scale (0–3), summed to yield a total score ranging from 0–21. Higher scores indicate more depressive symptoms with scores ≥7 meeting criteria for moderate or higher depressive symptoms. Current mood was assessed using the profile of mood states (POMS-A) [24], which is a psychological rating scale used to assess transient feelings of tension, depression, anger, fatigue, vigor and confusion. Self-efficacy was measured using the New General Self-Efficacy Scale (GSES) [25].

Participants also completed neuropsychological tasks: the Hopkins Verbal Learning Test Revised (HVLT-R) [26], measuring verbal learning over time; a digit span task [27]; and a matrix reasoning task [28] (baseline only).

### Diet compliance

Intake of foods recommended as part of the diet intervention was assessed using the Diet Compliance Score, a 10-item questionnaire developed for the study, which asked participants how many serves of the recommended food groups (fruit, vegetables, wholegrain and cereals, natural dairy products, lean protein, fish and other seafood, olive oil, nuts and seeds, olives or avocado and spices) they had consumed over the past 3 weeks. Higher scores indicated greater compliance with the recommended daily and weekly serving quantities. Intake of saturated fats and sugar was measured using the Dietary Fat and Sugar Screener (DFS) [17]. Participants were asked to rate how often they had consumed 26 food and drink items over the past year on a five-point category scale ranging from 1 = “Less than one time a month” to 5 = “Five or more times a week”. Intake of fruits and vegetables was measured was using the CM-700D Konica-Minolta Spectrophotometer, which measures the light the participant’s skin reflected and skin yellowness, estimating the quantity of flavonoids (chemicals from fruit and vegetables) in their diet [29] and demonstrated to predict plasma carotenoid levels following diet intervention [14]. Two readings from the palm of each hand were obtained with the spectrophotometer using the beta axis measure and averaged to create a single score [29].

### Clinical data

Body measurements (height and weight) were taken to calculate Body Mass Index (BMI). Current levels of physical activity were calculated using the International Physical Activity Questionnaire [30] and sleep quality using two items from the Pittsburgh Insomnia Rating Scale [31]. Participants were also asked the following questions related to medical history: whether they were a current smoker; whether they had any existing medical or psychological health conditions; and the names of any medications they were taking. Where additional consent was given, a urine and a finger-prick blood sample was taken to measure biological markers of inflammation (data not yet analysed and will be presented in more detail in a forthcoming publication).

### Sample size

Based on an estimated effect size of *d* = .80, alpha level = .01 (one-tailed as direction was hypothesised), power = .80, we estimated that we would require a total of n = 36 participants per group and therefore aimed to recruit 40 per group based on a 10% dropout rate.

### Randomisation and blinding

The randomization sequence was computer generated by an experimenter who was not involved in recruitment, assignment of participants to groups, or conducting baseline/Day 21 assessments. The randomization schedule was managed by the research assistant responsible for recruitment and enrolling eligible participants. At the conclusion of the baseline assessment, the participants were informed of their group allocation and those in the diet change group were provided the video and paper materials. By necessity of this design, the research assistants responsible for administering the questionnaires for the primary and secondary outcome measures were aware of the group allocations. However, several strategies reduced the risk of bias. Firstly, the outcome measures were either self-report, were administered by recording or computer (e.g. neuropsychological tasks) or objective (e.g. spectrophotometer), therefore these variables should not have been influenced by research assistants knowing group allocation. Secondly, only partial information was provided regarding the study hypothesis. Third, scoring of neuropsychological test scores and data entry was completed by an additional research assistant who was not involved in recruitment or testing.

### Data analysis

Data were screened and found to be suitable for parametric analysis, with the exception of the GSES which was log-transformed. We compared demographic, health measures, diet quality and psychological measures at baseline between participants with complete follow-up, using the chi- squared test for categorical data and *t* tests for continuous measures. The main analyses utilized ANCOVA with Group (diet change vs. habitual diet) as the between-subject factor, post-intervention scores as dependent variable and pre-intervention scores as covariate [32]. To examine whether intervention effects were maintained after 3 months, paired t-tests were conducted on the pre-intervention and 3 month follow up, and post-intervention and 3 month follow up scores on the DASS-21 depression subscale.

Cohen's *d* effect size values [33] were calculated based on the observed data. We calculated the Reliable Change Index (RCI) for each participant on the primary outcome measure [34] and the frequency of participants whose scores showed reliable positive change was compared between groups using chi-squared analysis. Intent- to-treat (ITT) analyses were conducted for the primary outcome measure (CESD score) and the secondary outcome measure for depression symptoms (DASS-21 depression subscale score), substituting post- intervention scores with pre-intervention scores for participants who were lost to post-intervention assessment [35]. We calculated difference scores (baseline and 3-weeks) for the two measures of depression symptoms (CESD-R and DASS-21-D) and the measures of diet quality (DFQS, DFS and spectrophotometer). Using these difference scores, multiple regression was performed to examine which diet variables were predictors of improvement in mood. Change in depression symptoms was the dependent variable (performed separately for CESD-R and DASS-21 depression scores) and the following diet variables were simultaneously entered; (i) change in processed food intake (DFS), (ii) change in intake of the recommended diet foods (DCS), and (iii) change in spectrophotometer scores.

## Results

**Fig 1:**
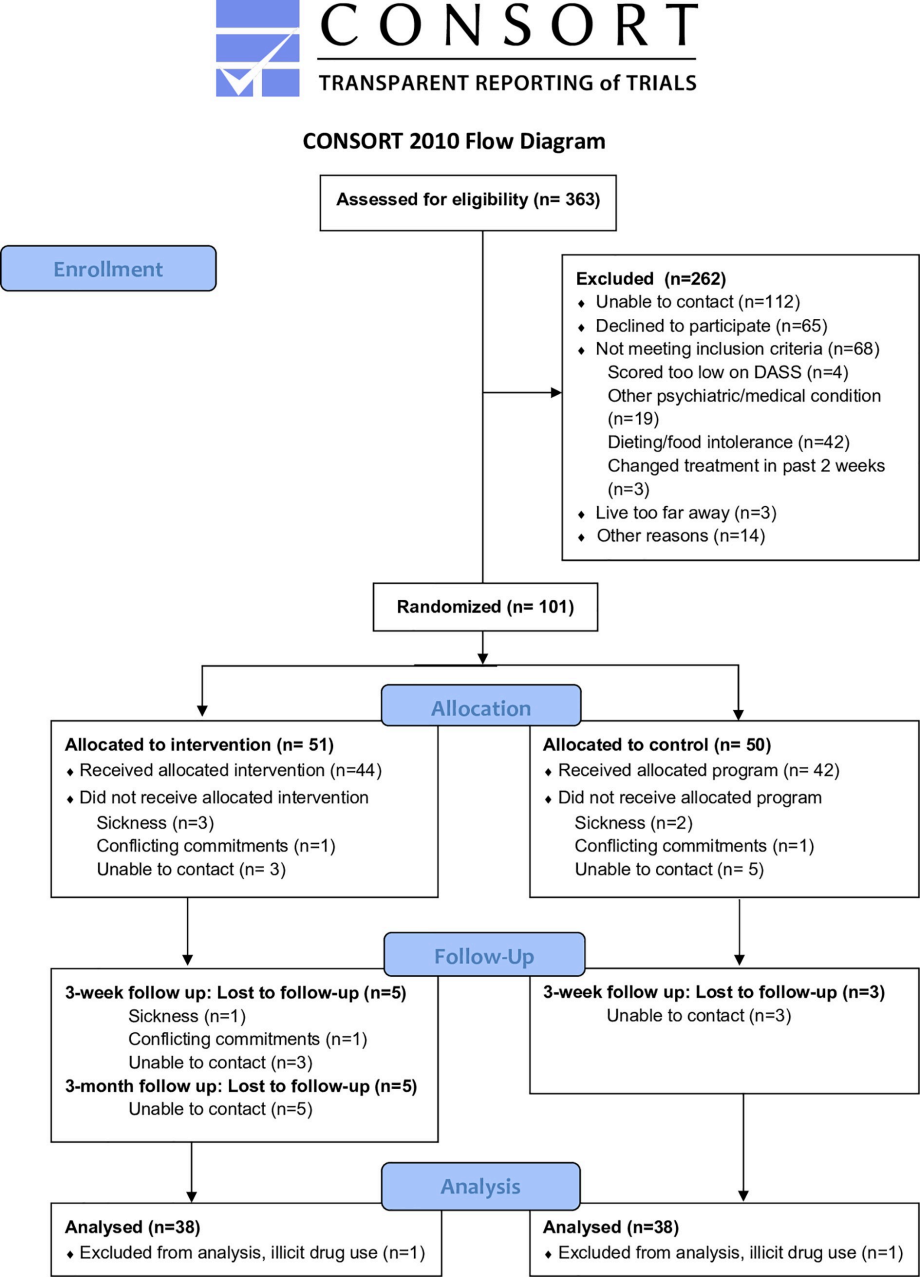
CONSORT flow chart.

We assessed 363 individuals for eligibility. Of these, 262 were excluded. We thus randomized 101 individuals with DASS-21-Depression scores of ≥ 7 on screening to the trial (DC group, n = 51; HD group, n = 50). Recruitment commenced on 08/05/2017 and final follow up assessment was conducted on 06/12/2018. Recruitment was ceased when we had recruited 39 per group because we had reached our anticipated sample size. At the time of completion of the study, 78 participants had completed testing at baseline and Day 21. An additional 2 were excluded due to illicit drug use during the duration of the intervention. Therefore, there were 38 participants in each group, all 38 were include in each analysis, and the analysis was conducted by original assigned groups. Fig 1 presents a CONSORT flow chart. Baseline characteristics of enrolled participants who completed both testing days are displayed in Table 1. The DC group and HD group did not differ on any of the demographic, health, diet or psychological questionnaire data at baseline (see Table 1; chi-squared, t-tests; all p's < .25).

**Table 1:** Baseline characteristics of individuals in the diet change (n = 38) and habitual diet (n = 38) groups.

	Diet Change (n = 38)	Habitual Diet (n = 38)
**Demographic variables**		
Gender (n, % female)	24, 63.0%	24, 62%
Age M(SD)	19.53 (2.05)	19.67 (2.80)
Intellectual function (Matrix Reasoning) M(SD)	5.84 (2.09)	6.05 (2.28)
**Health measures**		
BMI	22.07 (2.99)	22.39 (3.37)
Current Smoker (n, %)	4, 11%	6, 15%
Comorbid disorder (n, %)	8, 21%	11, 28%
Physical Activity (IPAQ Mets) M(SD)	6490 (3589)	5381 (3732)
Psychopharmacotherapy (n, %)	5, 13%	4, 10%
**Diet Quality**		
Diet Quality Screen M(SD)	48.58 (11.53)	46.44 (9.44)
Diet Fat and free Sugar Screener M(SD)	68.58 (16.43)	64.62 (12.93)
Spectrophotometry M(SD)	15.83 (1.65)	15.89 (1.70)
**Depression symptoms**		
CESD-R	20.56 (2.04)	20.28 (2.59)
DASS-21-Depression	7.18 (0.84)	7.03 (0.94)

We used the DASS-21 depression subscale as a screening tool for brevity compared to the CESD-R. Although a score ≥7 on the DASS-Depression scale was a criteria for enrolment, scores at baseline testing on Day 1 were slightly different compared to screening. Table 2 shows DASS-21-Depression scores at baseline for the DC and HD group divided into severity.

**Table 2:** Level of severity on the DASS-21 depression subscale at baseline for those in the diet change and habitual diet groups.

Severity labels:		Normal (0–4)	Mild (5–6)	Moderate (7–10)	Severe (11–13)	Extremely Severe (14+)
Diet Change Group	n %	12 31.6%	9 23.7%	5 13.2%	7 18.4%	5 13.2%
Habitual Diet Group	n	16	8	4	5	6
	%	41.0%	20.5%	10.3%	12.8%	15.4%

### Primary outcome: Depression symptoms

**Fig 2:**
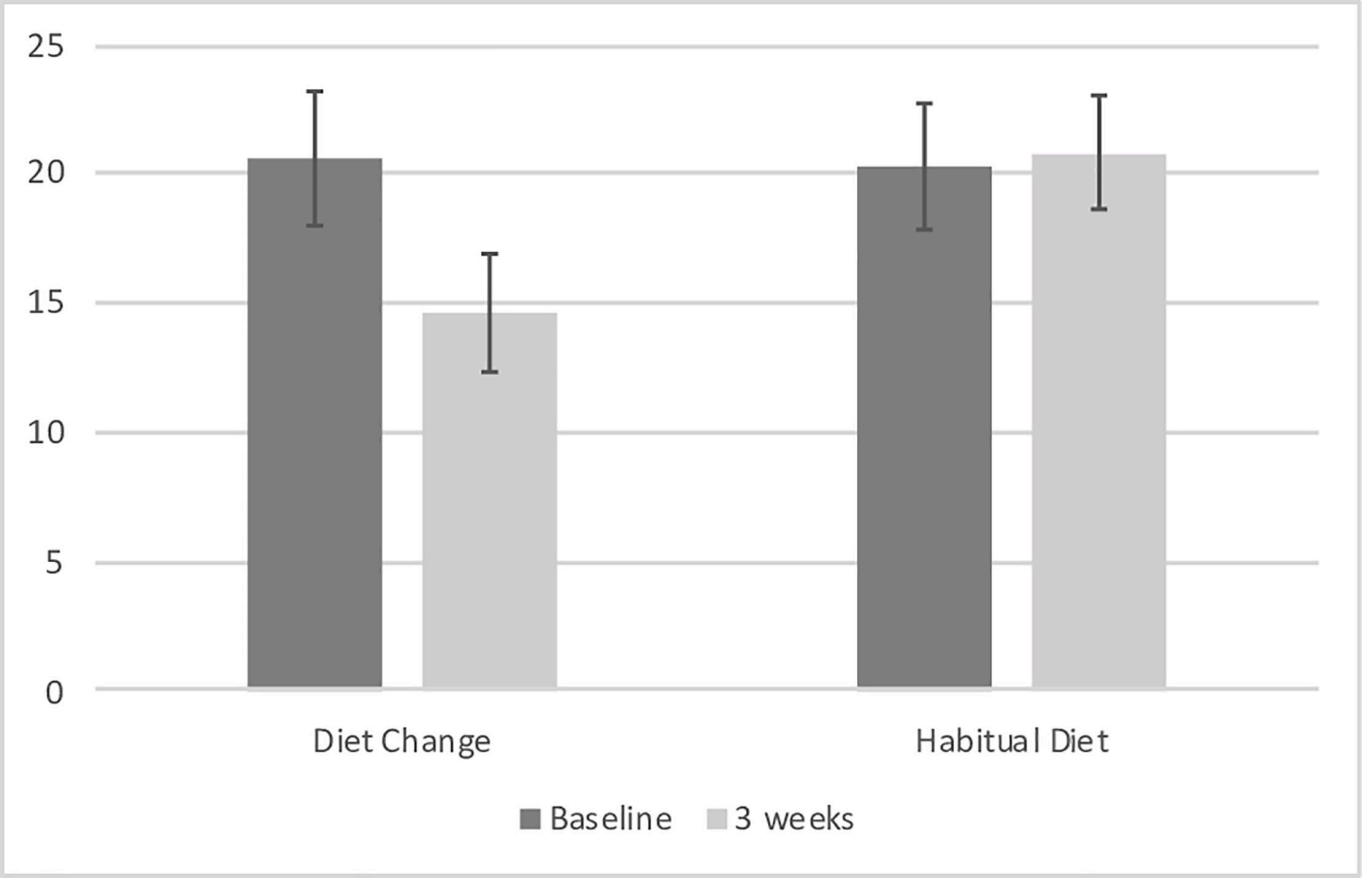
Center for Epidemiological Studies Depression scale—Revised scores for diet change (n = 38) group were significantly lower than the habitual diet (n = 38) group following 3 weeks of diet improvement, controlling for baseline scores (effect size: Cohen’s d = 0.65).

The average CESD-R score for the DC group improved from the elevated range (i.e. >16) to the no clinical significance range, but remained elevated in the HD group across baseline and day 21 (see Fig 2). This difference was significant, with the DC group having significantly lower CESD-R scores on Day 21 compared to the HD group, controlling for baseline CESD-R scores (F[1,75] = 7.792, *p* = .007, Cohen’s *d* = 0.65; see Table 3). When the ANCOVA was rerun, additionally controlling for age, gender, physical activity and baseline BMI, the significant group difference at Day 21 remained (F[1,71] = 7.091, *p* = .010).

**Table 3:** Mean (±standard error) scores for diet change and habitual diet groups on the primary and secondary outcome measures, as well as results of ANCOVA comparing groups at Day 21 while controlling for Day 1 scores.

Measure	Diet Change Group		Habitual Diet Group		F	*p*
	Day 1 M (SE)	Day 21 M (SE)	Day 1 M (SE)	Day 21 M (SE)		
**Depression symptoms**						
CESD-R	20.56 (2.04)	14.62 (1.77)	20.28 (2.59)	20.81 (2.24)	7.792	.007
DASS-Depression	7.18 (0.84)	4.37 (0.64)	7.03 (0.94)	6.59 (0.92)	10.104	.002
DASS-Anxiety	6.26 (0.78)	3.37 (0.56)	4.92 (0.54)	4.36 (0.63)	5.262	.032
DASS-Stress	7.66 (0.79)	4.82 (0.56)	6.44 (0.72)	6.51 (0.64)	8.877	.004
**Current mood**						
POMS-Anger	1.00 (0.32)	0.89 (0.28)	1.77 (0.45)	2.41 (0.56)	3.691	.059
POMS-Depression	2.26 (0.43)	1.76 (0.47)	3.41 (0.73)	2.97 (0.65)	0.554	.459
POMS-Confusion	3.08 (0.46)	2.08 (0.40)	3.56 (0.55)	3.08 (0.56)	1.668	.201
POMS-Tension	3.76 (0.51)	2.87 (0.54)	3.13 (0.48)	3.13 (0.45)	0.576	.450
POMS-Vigour	4.84 (0.65)	4.92 (0.58)	4.54 (0.48)	4.59 (0.49)	0.065	.800
POMS-Fatigue	7.45 (0.64)	6.18 (0.62)	7.18 (0.72)	7.10 (0.66)	1.504	.224
**Self-efficacy**						
GSES	20.16 (0.77)	19.32 (0.41)	20.01 (0.55)	20.58 (0.62)	3.217	.077
**Memory**						
HVLT-linear	1.9 (0.1)	2.2 (0.1)	1.8 (0.1)	1.9 (0.1)	2.822	.097
HVLT-quadratic	-0.6 (0.1)	-0.8 (0.1)	-0.8 (0.2)	-0.9 (0.2)	0.394	.532
HVLT-% recall	95.56 (1.59)	94.97 (1.65)	93.9 (1.7)	96.2 (1.6)	0.478	.491
**Diet**						
DCS	48.58 (1.87)	65.82 (1.77)	46.44 (1.51)	42.33 (1.42)	122.786	.000
DFS	68.58 (2.67)	45.13 (1.42)	64.62 (2.07)	60.69 (1.91)	51.969	.000
Spectrophotometry	15.82 (0.27)	16.05 (0.29)	15.88 (0.27)	15.76 (0.27)	2.978	.089

CESD-R, Center for Epidemiological Studies Depression scale-Revised; DASS, Depression Anxiety and Stress Scale; POMS, Profile of Mood States; GSES, General Self-Efficacy Scale; DCS, Diet Compliance Score; DFS, Dietary Fat and free Sugar screener.

### Secondary outcomes

#### Depression symptoms

**Fig 3:**
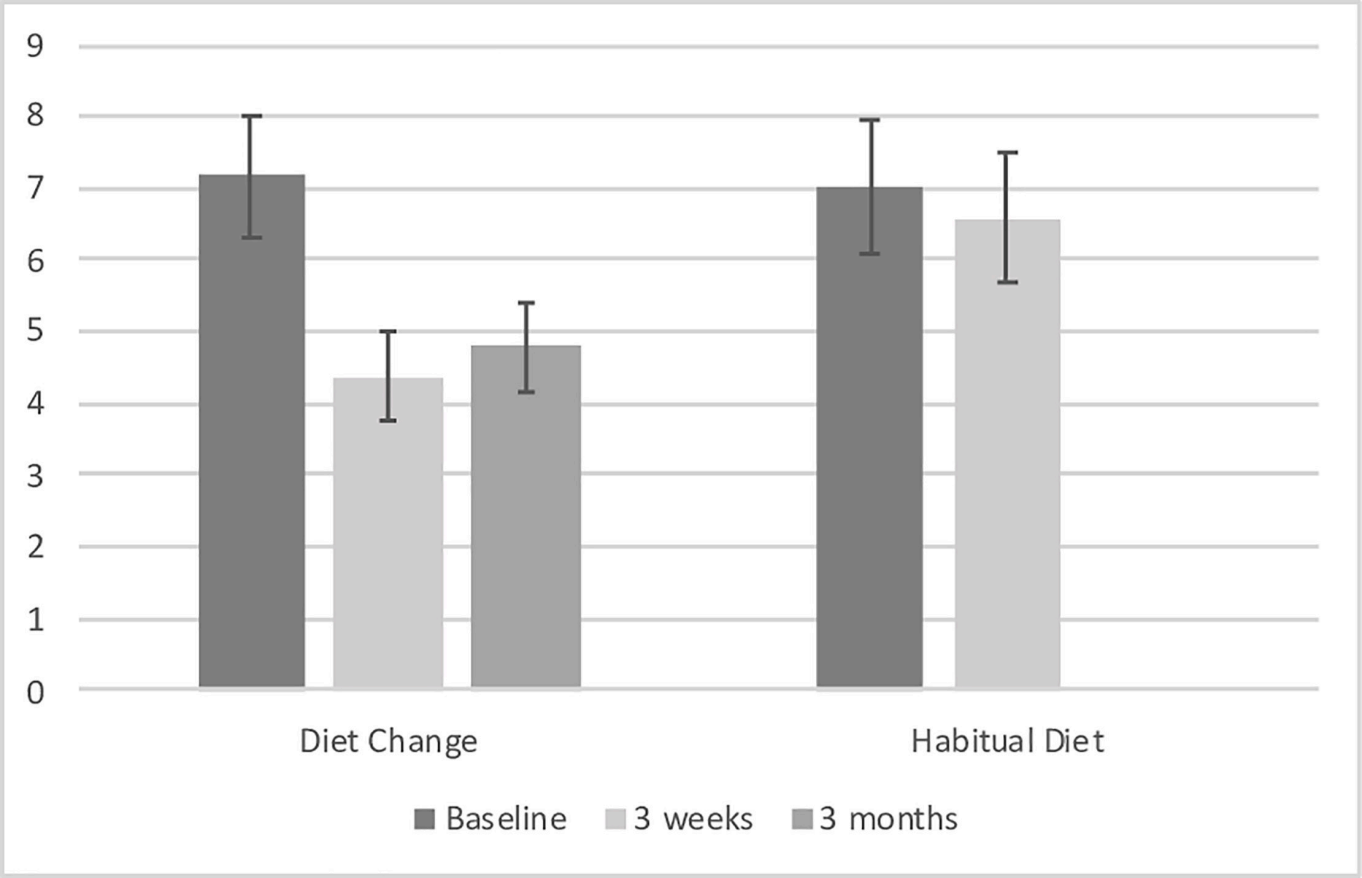
DASS-21 Depression subscale scores for diet change (n = 38) group were significantly lower than the habitual diet (n = 38) group following 3 weeks of diet improvement, controlling for baseline scores (effect size: Cohen’s d = 0.65) and remained significantly lower than baseline at 3 month follow up for the diet change group (n = 33).

The average DASS-21-Depression score for the DC group improved from the moderate severity range (i.e. scores 7–10) to the normal range (i.e. scores 0–4), but remained stable in the moderate severity range for the HD group across baseline and Day 21 (see Fig 3). This improvement was significant, with the DC group having significantly lower DASS-21-Depression scale scores on Day 21 compared to the HD group, controlling for baseline DASS-21-Depression scores (F[1,75] = 10.104, *p* = .002, Cohen’s *d* = 0.75). When the ANCOVA was rerun, additionally controlling for age, gender, physical activity and baseline BMI, the improvement at Day 21 remained significant (F[1,71] = 8.165, *p* = .006).

#### Additional mood measures

The DC group had significantly lower DASS-21-Anxiety scale scores on Day 21 compared to the HD group, controlling for baseline DASS-21-Anxiety scores (F[1,75] = 5.262, *p* = .0325, Cohen’s *d* = 0.54). The DC group had significantly lower DASS-21-Stress scale scores on Day 21 compared to the HD group, controlling for baseline DASS-21-Stress scores (F[1,75] = 8.877, *p* = .004, Cohen’s *d* = 0.70).

There were no significant differences between groups for current mood assessed using the POMS-A subscales. However, the DC group displayed a trend toward lower POMS-Anger ratings on Day 21 compared to the HD group, controlling for baseline POMS-Anger ratings (F[1,75] = 3.691, *p* = .059). There was no significant difference between groups with respect to self-efficacy.

#### Memory

There were no significant group differences on objective (word list learning) memory performance (linear learning curve, quadratic learning curve or percent recall) over the duration of the intervention (*p*’s >.05), even when controlling for baseline intellectual functioning (assessed using Matrix Reasoning).

We asked participants whether they had experienced a stressful event during the 3 weeks of the intervention. There was no significant difference between groups, with 22 (58%) of participants in the DC group and 21 (54%) of participants in the HD group reporting a stressful event occurring during that period (Chi-squared = 0.016, *p* = .898). No participants reported ceasing their medications. Two individuals in the HD group started taking psychopharmacological medication over the 3 weeks. The primary outcomes (CESD-R and DASS-21-Depression) remained significantly different between groups even when re-analysed excluding these two individuals. There was no significant difference between groups for BMI, physical activity or of poor health (e.g. suffering colds) (*p*’s>.48).

### Intent to treat

For the intent to treat (ITT) analysis, pre-intervention scores were used as post-intervention scores for the 11 participants who did not complete the Day 21 assessment. ANCOVA results obtained from ITT analyses showed that the between-group effects remained significant for both the CESD (F [1,84] = 7.414, *p* = .008) and the DASS-21-Depression subscale (F [1,84] = 9.110, *p* = .003).

### Reliable change

A significantly greater frequency of reliable change was demonstrated in the diet change group compared to the habitual diet group (*x*^2^ = 3.34, *p* = .034), with 10 individuals in the diet change group and 4 individuals in the habitual diet group showing reliable change on the CESD-R.

### Compliance with diet recommendations

The DC group showed a significant increase in consumption of the recommended foods (DCS scores) from baseline to 3 weeks compared to the HD group (F(1, 75) = 122.786, *p* = .000, Cohen’s *d* = 2.58). This remained significant after controlling for age, gender, baseline BMI and baseline physical activity (F(1, 71) = 124.183, *p* = .000). A greater change in spectrophotometer scores (representing increased fruit and vegetable intake) within the DC group was associated with a greater change in DCS scores (representing compliance with diet recommendations), *r*(38) = .435, *p* = .003. This relationship was not present in the control group, *r*(38) = .115, *p* = .242.

Conversely, the DC group showed a significant reduction in consumption of foods high in saturated fat and refined sugar (DFS scores) from baseline to 3 weeks compared to the HD group (F(1, 75) = 51.969, *p* = .000, Cohen’s *d* = 1.67). This remained significant after controlling for age, gender, baseline BMI and baseline physical activity (F(1, 71) = 47.169, *p* = .000).

To examine which diet variables best predicted change in depression scores over the intervention, CESD-R and DASS-21 depression scores were entered as the dependent variable in two separate analyses, with change in i) processed food intake (DFS), ii) recommended food intake and iii) spectrophotometer scores, as predictor variables.

With the CESD-R as the dependent variables, the overall regression model was significant (F(3,34) = 4.822, *p* = .007) and explained 23.7% of the variance (adjusted R-squared). Change in processed food intake uniquely explained 15.8% of the variance (beta = 0.219, *p* = 0.009). Neither change in intake in recommended diet foods nor change in spectrophotometry score made a unique contribution, though the latter approached significance (beta = .372, *p* = .066).

With the DASS-21 depression subscale score as the dependent variable, the overall regression model was significant (F(3,34) = 2.951, *p* = .046) and explained 13.7% of the variance (adjusted R-squared). No unique contribution was made by any variable individually.

### Three-month follow-up

Thirty-three individuals were able to be contacted via telephone for 3-month follow up. As this was done via telephone, only minimal data were collected. Paired t-tests revealed that DASS-21 depression scores at 3 months (M = 4.79, SD = 3.36) remained significantly lower than at baseline (M = 7.73, SD = 4.87), *t*(32) = 3.68, *p* = .001, and did not differ significantly from scores at 3 weeks (M = 5.03, SD = 4.36), *t*(32) = 0.63, *p* = .534. Overall, this indicates that intervention effects were maintained for the diet change group at 3-month follow up. Of the 33 individuals who were able to be contacted, 7 (21.2%) said they had maintained the diet, 19 (57.6%) reported they had maintained some aspects of the diet and 7 (21.2%) reported they had not maintained the diet. There was no difference in depression outcomes between these 3 groups (chi-square >.05).

## Discussion

This study examined for the first time whether a brief diet intervention could improve depression symptoms in young adults. In line with our hypotheses, we found significant reductions in depressive symptoms on the primary outcome measure, the CESD-R, for those in the diet change group compared to the habitual diet control group, with a moderate effect size of 0.65. Similarly, significant improvements were observed for the secondary outcome measure of depressive symptoms, the DASS-21 depression subscale, with a moderate effect size of 0.75. Also in keeping with our hypotheses, degree of compliance with diet as associated with improvements in depressed mood for both of the above measures.

The results of this RCT provide support for improving diet as a useful adjunct treatment to reduce depressive symptoms. Note here the relationship with prospective studies. In light of these attenuating findings, the authors suggested the field would benefit from randomised controlled trials (amongst other recommendations). The data support findings of the SMILES trial, which showed that a 12-week diet intervention resulted in significant reduction in depression symptoms [11]. Although the current study obtained a lower effect size than the SMILES trial, there were two main points of difference that we hypothesise may have contributed to this. First, the primary outcome measure in the SMILES trial was the MADRS, which is a clinician administered rating scale. Due to the expertise of the clinicians administering the scale, it may be more sensitive. In support of this interpretation, similar effect sizes to the present study were reported in the SMILES trial using the self-report scale (Cohen’s *d* = 0.632 for HADS depression subscale).

The current study was performed in young adults with low mood, with a score ≥ 7 (corresponding to moderate levels of depression symptoms) on the DASS-Depression scale a criteria for enrolment. However, there was some fluctuation in scores between enrolment and the baseline testing, with 12 participants in the DC group and 16 participants in the HD group who scored within the normal range at baseline. If anything, this may have meant the effect of the diet intervention was less pronounced, as the overall severity was not as high as the lower baseline severity of the sample may have meant that improvements were harder to detect. Nevertheless, we emphasize that it is even more noteworthy that an effect was obtained under those circumstances.

Improvements were also noted on the anxiety and stress subscales of the DASS-21, with overall moderate effect sizes. Importantly, there were no differences between groups for rate of stressful events throughout the duration of the intervention. This finding is also consistent with the SMILES trial, which showed the diet intervention improved anxiety symptoms using the Hospital Anxiety and Depression Scale [11]. There are few studies investigating the ability for diet to remediate anxiety symptoms in otherwise healthy adults, and the observational literature is not as well established as the literature regarding diet and depression, but these findings suggest this is a field of research worth pursuing.

To our knowledge, there is only one other randomized controlled trial of a diet intervention in young adults, which included mood as an outcome. There was no difference in depressed or anxious mood between a diet change and a ‘no change’ control group in females aged 19–30 [12], however, the measure employed in this study was the POMS, which measures current mood. Specifically, the scale asks respondents to rate how much they are “feeling” particular emotions “right now”, which may be more transient than the depression symptoms tracked in questionnaires (e.g. the CESD-R, DASS and HADS) and diagnostic interviews (e.g. the MADRS). This interpretation is supported by the fact that current mood assessed using the POMS-A in the current study did not differ between the groups. And is also in keeping with the SMILES trial, which found improvement following the diet intervention on the MADRS and HADS, but not the POMS [11].

One of the most interesting findings is the fact that diet change was feasible in this population. As the participants were young adults and university undergraduate students, we anticipated several potential barriers such as the perceived cost of the diet, the time demands of preparing food and/or reliance on others for food preparation (particularly if they lived at home). Additionally, the participants were recruited based on self-reported symptoms of depression. We anticipated that the symptoms of depression, including low energy, reduced motivation and apathy, would present as barriers to eating well. Despite these factors, there was a significant increase in the recommended foods and decrease in processed foods for the diet change group but not the habitual diet group. Furthermore, within the diet change group, increase in recommended foods was associated with spectrophotometer readings. This provides objective evidence to support the participants’ self-reported compliance with the diet [14]. Regardless of the impact of diet change on depressed mood, the physical health benefits of a Mediterranean style diet are well established [36].

Regardless of which way the causal arrow runs, there is strong evidence that people with depression typically have unhealthy dietary habits [2, 7]. Individuals diagnosed with mental illness experience high rates of morbidity and mortality as a result of poor physical health and unhealthy lifestyle behaviours. Therefore, recommending that they improve their diet may be done with the aim of improving mood or improving physical health. Even in the general population, adherence to diet advice is typically very poor, with over 80% of Australians reporting that they do not comply with dietary recommendations [37]. As a result, there is substantial nihilism regarding the ability to change people’s diets. The current study simply provided a brief 13-minute video, paper resources and minimal phone support. The fact that this relatively low-cost intervention can result in a population of young adults adhering to diet recommendations is very promising. Furthermore, it is important to consider that participants in the current study did not need to adhere strictly to the diet recommendations to derive benefit. This is consistent with previous studies that have shown substantial risk reductions with moderate adherence to healthy diet patterns, and indeed that maximum adherence provides almost no extra benefit [38]. This is important clinically, as health professionals can adopt a less strict harm reduction approach.

We note that, in addition to providing the diet recommendations, we additionally provided substantial resources to troubleshoot dealing with cravings, psychosocial influences on eating and employing cost-saving measures. We speculate whether this was a key factor in ensuring adherence, however this remains to be tested in future studies. The intervention in this study was quite brief however previous studies have shown that individuals with Major Depressive Disorder [11] and even young people with first-episode psychosis [39], can adhere to a prescribed diet over longer durations. This study therefore contributes to a body of research suggesting that despite many perceived barriers, nutritional intake can be modified in youth with mental health issues.

Although the DC group demonstrated a significant increase in the recommended foods and a significant decrease in processed foods, we unexpectedly did not observe a correlation between an increase in one and a decrease in the other. We suggest this is perhaps because some individuals focused on increasing intake of the healthier foods whereas others focused more on decreasing intake of processed foods. This afforded the opportunity for analyzing which dietary components had the strongest relationship with change in depressed mood. Regression analysis suggested that reduction in processed foods contributed the most variance to improvement in depression symptoms over the course of the intervention. This would suggest that, in addition to recommending individuals partake in a healthy diet, an important factor is to reduce intake of processed foods. The recommendations provided in this study included avoiding foods that come in a package with multiple ingredients, foods with more than 10g sugar per 100g, and provided specific examples including soft-drinks, chocolates, sweets and fried takeaway foods.

Telephone follow up at 3-months following the intervention revealed that, for the 33 participants who were able to be contacted, the improvements in depression symptoms had been maintained over this period and remained significantly reduced compared to baseline. The telephone call was brief to maximise compliance, however this meant we obtained little data regarding whether diet was maintained over this period. Most (57.6%) reported that they had maintained some aspects of the diet, whereas 21.2% reported that they had and 21.2% reported that they had not. No significant difference was found between these groups in terms of depression outcomes, however this may have been due to either lack of sufficient power or lack of sensitivity in the diet question. Future research would benefit from face-to-face attendance of follow-up to obtain more data or at least administration of food frequency questionnaires via telephone.

There are several limitations to this study. First, the sample was undergraduate university students, therefore further research would be needed to determine if this type of intervention would be appropriate for young adults more generally. By necessity of the testing arrangements and funding limitations, the research assistants who administered the questionnaires also provided the diet resources. There was little interpretation required of the research assistants since mood measures and diet measures were self-report and the data entry was performed by a different research assistant who was blind to the conditions of the participants, therefore it seems unlikely this would have affected the results. Nevertheless, in future studies it would be ideal to have a separate research assistant administer the mood questionnaires and diet resources.

Another limitation of the current study is the lack of an active control group as a comparison. However, there are difficulties in determining an appropriate active control. An alternative diet manipulation such as a low-fat diet would be problematic as they may similarly result in a reduced in processed food intake, or asking participants to consume a less-healthy diet would be unethical if this were to worsen their mood symptoms. Another option would be to include a control group receiving psychological therapy, however in terms of translating the findings to clinical practice, we would not be advocating that diet intervention should replace psychological therapy. In fact, the study was conducted concurrently with treatment as usual. Furthermore, meta-analysis of RCTs conducted in older adults found no difference between studies using an active versus an inactive control group [9]. Thus, the findings support what we would recommend as clinical practice; that there is evidence to support advising young adults who present with depressive symptoms to improve their diet, *as an adjunct to* pharmacological and psychological interventions. There remains the possibility that either the provision of the food hampers or the slightly higher degree of examiner contact in the diet intervention group (i.e. 2x 5-minute phone calls during the 3 weeks) could account for the differences between groups. However, the finding that change in diet (assessed via both self-report and objective spectrophotometry measures) was significantly related to improvement in depression symptoms (as measured by both the CESD-R and the DASS) provides support for our hypotheses that it was the change in diet per se that resulted in improved depression symptoms.

The current intervention involved such a small degree of face-to-face contact and very little cost or risk, thus there are few downsides to adopting this approach to improving mood. Conversely, there is a lot to gain not just in terms of improvements to mood but also in enhanced physical health outcomes. We hope these findings provide the impetus for future research examining whether the recommended diet can be sustained over longer durations in this population and whether the effects on depression symptoms are maintained.

## Supporting information

S1 File Trial study protocol. (PDF)

S2 File CONSORT checklist. (DOC)

S3 File Data file. (XLSX)
