# Supplementary material for: A brief diet intervention can reduce symptoms of depression in young adults – A randomised controlled trial
Source: PLoS One. 2019 Oct 9;14(10):e0222768. doi: 10.1371/journal.pone.0222768 (PMC6784975; doi:10.1371/journal.pone.0222768)
Supplement: S1 File — (PDF) [file pone.0222768.s001.pdf]

MACQUARIE University

Diet, mood and cognition study

Protocol Number:

Principal Investigator: Prof Richard J. Stevenson; (02) 9850 8098

Signature: Date:

Protocol Authors: Heather Francis, Richard Stevenson, Edwin Lim

Protocol Version # 1

Protocol Date:

Proprietary Notice (if applicable)

Ethics Statement:

The study will be conducted in accordance with the *National Statement on Ethical Conduct in Human Research* (2007), the *CPMP/ICH Note for Guidance on Good Clinical Practice* and consistent with the principles that have their origin in the Declaration of Helsinki. Compliance with these standards provides assurance that the rights, safety and well-being of trial participants are respected.

Contents

Summary 3

1. BACKGROUND AND INTRODUCTION 4
   - 1.1. DISEASE/PROPOSED INTERVENTION BACKGROUND 4
   - 1.2. RATIONALE FOR PERFORMING THE STUDY 4
2. HYPOTHESIS 4
3. STUDY OBJECTIVES 5
   - 3.1. PRIMARY OBJECTIVES 5
   - 3.2. SECONDARY OBJECTIVES 6
4. STUDY DESIGN 7
   - 4.1. DESIGN 7
   - 4.2. EXPECTED PARTICIPANT NUMBERS 7
   - 4.3. DURATION OF THE STUDY 7
   - 4.4. ENDPOINTS 7
   - 4.5. CENTRES 8
5. STUDY PARTICIPANTS 8
   - 5.1. INCLUSION CRITERIA 8
   - 5.2. EXCLUSION CRITERIA 8
6. STUDY PROCEDURES 9
   - 6.1. STUDY FLOW CHART 9
   - 6.2. INVESTIGATION PLAN 8
   - 6.3. STUDY PROCEDURE RISKS 13
   - 6.4. PARTICIPANT RECRUITMENT AND SCREENING 13
   - 6.5. PARTICIPANT ENROLMENT 14
   - 6.6. INFORMATION AND CONSENT 14
   - 6.7. RANDOMISATION PROCEDURE 14
   - 6.8. END OF STUDY TREATMENT/WITHDRAWAL PROCEDURE 14
   - 6.9. PATIENT WITHDRAWAL 14
7. OUTCOMES 14
   - 7.1. DEFINITION OF OUTCOMES 14
8. STATISTICAL CONSIDERATIONS 15

8.1. SAMPLE SIZE OR POWER CALCULATION 15
8.2. PROVIDE A DETAILED ANALYSIS PLAN 15
9. DATA COLLECTION 15
9.1. PARTICIPANT REGISTRATION 15
9.2. FORMS AND PROCEDURE FOR COLLECTING DATA 16
10. QUALITY CONTROL AND ASSURANCE 17
10.1. CONTROL OF DATA CONSISTENCY 17
10.2. AUDITS 17
10.3. PROTOCOL AMENDMENTS 18
11. ETHICS 18
11.1. INVESTIGATOR AUTHORISATION PROCEDURE 18
11.2. PATIENT PROTECTION 18
12. SAFETY 18
12.1. ADVERSE EVENT REPORTING 18
12.2. SERIOUS ADVERSE EVENT REPORTING 18
13. BLINDING AND UNBLINDING 18
14. CONFIDENTIALITY AND STORAGE AND ARCHIVING OF STUDY 19
15. TRIAL SPONSORSHIP AND FINANCING 19
16. REFERENCES 19
17. APPENDICES 20

Summary

Study title: Diet, mood and cognition study

Primary objective: To examine whether a dietician designed 3-week program (as an adjunct to medical treatment as usual) results in improvements in self-reported mood.

Secondary objectives:

To examine whether the diet intervention also:

1) Reduces levels of inflammatory biomarkers: i) proinflammatory cytokines and ii) altered kynurenine pathway metabolites with reduced QA and increased KA.
2) Improves performance on hippocampal dependent neuropsychological tasks

Study design: Randomised-controlled trial mixed design

Planned sample size: 80 total (40 per group)

Selection criteria: elevated (>7) scores on the Depression, Anxiety and Stress Scale-21 Depression subscale (DASS-21-D); Score > 57 on the Dietary Fat and Sugar Screener (DFS); no history of metabolic, cardiovascular or eating disorders; not pregnant, not currently dieting, no religious or medical factors affecting adherence to diet. If receiving antidepressant medication or therapy, on the same treatment for at least 2 weeks before study participation.

Study procedure: Participants will attend 2 sessions, 3 weeks apart (baseline & day 21). In both sessions, participants will complete mood measures, neuropsychological tasks, food questionnaires, body measurements and photospectrometry. Participants are randomly assigned to diet intervention or no change group. Those in the diet intervention will receive dietician designed advice, those in the no change group will continue diet as usual, but receive the same diet advice at the conclusion of the study (after day 21 measurements).

Duration of the Study: 24 months

1. BACKGROUND AND INTRODUCTION

1.1. DISEASE/PROPOSED INTERVENTION BACKGROUND

There has been a rapid global shift in dietary composition, from diets high in complex carbohydrate and fibre, to the Western diet, high in saturated fat, sugar and processed foods. This was highlighted recently by Australia's largest ever diet survey, the CSIRO Healthy Diet Score, 2016, which showed of 86,611 respondents, only 20% were compliant with Australian Diet Guidelines (Hendrie et al, 2016). Consumption of Western diet has been accompanied by considerable adverse health consequences, with the effects on the brain now increasingly recognised.

Unhealthy diet patterns are associated with increased risk of depression (e.g. a meta-analysis of 20 observational studies showed healthy diet was associated with reduced odds of depression; Lai et al, 2014). But these studies were correlational so a cause-effect relationship could not be proved, therefore experimental manipulation of diet is the next step. This study aims to investigate whether improving diet will cause improvements in individuals with elevated symptoms of depressed mood. This is the first step to establishing the basic science required to justify studies in a clinical population. Given diet is one of the few modifiable risk factors for depression, this would offer a low-cost, low-risk, evidence based therapeutic and preventative strategy. However, treatment studies investigating diet as an effective adjunct treatment for depression are scarce and none have investigated physiological mechanisms underlying the relationship (see Orygen Research Bulletin, Issue 3 for review).

A likely mechanism by which diet could impact on mood is inflammation. High intake of saturated fat, refined starches and sugar promotes inflammation, whereas high intake of fruit and vegetables, whole grains and omega-3 fatty acids reduces inflammation (Kiecolt-Glaser, 2010). Inflammation is part of the body's immune response, causing the individual to feel weak, tired, slow and antisocial. This is beneficial in helping fight infection, but when chronic may cause depression (i.e. people with depression have raised inflammatory markers and inflammatory diseases are associated with higher rates of depression; Howren, 2009).

1.2. RATIONALE FOR PERFORMING THE STUDY

Treatments for depression typically target the neurotransmitter serotonin. Inflammation is associated with depression and actually decreases serotonin being made from tryptophan along the kynurenine pathway. But serotonin is only one brain chemical that can be affected by this pathway. Inflammation increases excitotoxic quinolinic acid (QA) and reduces neuroprotective kynurenic acid (KA), and these brain chemicals are altered in the same direction in depression (Myint et al, 2007). But this does not prove a cause and effect relationship, therefore, experimentally manipulating inflammation and examining effect on mood is the next logical step. However, there are risks with increasing inflammation, therefore decreasing inflammation through diet offers a low-risk paradigm for investigating a cause-effect relationship between the kynurenine pathway and mood.

2. HYPOTHESES

Design summary: This study will recruit individuals with self-reported depressed mood on a brief screening measure. They will be randomly assigned to one of two groups: diet change (DC) or maintenance of habitual diet (HD). They will attend 2 sessions (pre- and post-intervention).

We hypothesise that, compared to the diet as usual group, those on the healthy diet for 3 weeks will show:
1) will reduce depressed mood;

2) will reduce biomarkers of inflammation (as specified above);
3) will improve performance on hippocampal dependent neuropsychological tasks
3) levels of depressed mood will be correlated with changes in inflammatory biomarkers.

The benefits of including analysis of markers of inflammation in this study are two pronged: 1) to provide evidence of a mechanism underlying any beneficial effects of healthy diet on mood and 2) to experimentally manipulate levels of inflammation (via diet) to contribute to understanding the causal relationship between the kynurenine pathway and mood.

3. STUDY OBJECTIVES

3.1.PRIMARY OBJECTIVES

To examine whether a dietician designed 3-week program (as an adjunct to medical treatment as usual) results in improvements in self-reported depressed mood.

3.2.SECONDARY OBJECTIVES

To examine whether the diet intervention also:

1) reduces levels of inflammatory biomarkers: i) proinflammatory cytokines and ii) altered kynurenine pathway metabolites with reduced QA and increased KA.
2) Improves performance on certain other cognitive tasks

4. STUDY DESIGN

4.1. DESIGN

A randomised parallel groups trial assessing the effects of a 3-week diet change (DC) or no diet change (NC) on depressed mood and biomarkers of inflammation.

4.2. EXPECTED PARTICIPANT NUMBERS

This study will use 80 young adults (n=40 per group). Sample size was based on ability to detect relationships between the most conservative key dependent variables; diet and mood. McMillan et al (2011) showed a 10 day Mediterranean diet improved mood (d=1.34) and a recent RCT by Jacka et al. (2017) showed a 6 month diet intervention improved mood (d=1.16). There are no other whole-diet change trials to base our estimations on, therefore, we have conservatively estimated an effect size of .80, alpha level.01 (one-tailed as direction is hypothesised), power.80, a total of n = 36 participants per group is required (we aim for 40 per group based on a <10% dropout rate in the Jacka at al, 2017, study). Intention to treat analysis will be carried out for non-compliance.

4.3. DURATION OF THE STUDY

The study will run for approximately 24 months, running between April 2017 to April 2019

4.4. ENDPOINTS

PRIMARY ENDPOINTS

- Change from baseline performance on self-reported depressed mood following the 3-week diet intervention
- Change from baseline performance on neuropsychological tasks following the 3-week diet intervention
- Change from baseline levels of biomarkers of inflammation in blood and urine samples following the 3-week diet intervention

SECONDARY ENDPOINTS

- Correlation between biomarkers of inflammation and self-reported mood
- Correlation between biomarkers of inflammation and performance on neuropsychological tasks
- Correlation between reduction of processed foods (DFS) and biomarkers of inflammation
- Correlation between increase in fruit and vegetable intake (spectrophotometry) and biomarkers of inflammation

4.5. CENTRES

All testing sessions for all participants will be held in Room 505, Building C3B, Macquarie University. Urine sample collection will occur in an adjacent bathroom on the same floor. For those who

additionally consent to blood analysis, sample collection will be undertaken at Douglas Hanly Moir, Macquarie Private Hospital.

5. STUDY PARTICIPANTS

5.1. INCLUSION CRITERIA

Aged 17-35, elevated (>7) scores on the Depression, Anxiety and Stress Scale-21 Depression subscale (DASS-21-D); Score > 57 on the Dietary Fat and Sugar Screener (DFS).

5.2. EXCLUSION CRITERIA

Pregnant women, currently dieting, history of eating disorders or metabolic disease(s), history of psychological illness other than depression or anxiety, medical condition that could be adversely affected by diet change, poor proficiency in English, which may interfere with ability to understand study requirements, self-reported current or recent illicit drug use or alcohol use, sickness in the past week. If receiving antidepressant medication or therapy, on the same treatment for at least 2 weeks before study participation.

6. STUDY PROCEDURES

6.1. STUDY FLOW CHART

Day	Event	Duration (minutes)
**Day 1 (baseline)**	**Both groups**<br>Online: Medical history, International Physical Activity Questionnaire, Pennsylvania Insomnia Rating Scale 2, General Self-Efficacy Scale, CESDR, DASS-21, Profile Of Mood States, DFS, Diet Compliance Survey, Eating Habits Questionnaire (Diet Change group only)<br>C3B 503: Urine sample, Finger prick blood sample, Anthropometric measures, photospectrometry, Hopkins Verbal Learning Test, Digit Span Forwards, Matrix Reasoning<br>Douglas Hanly Moir: Serum blood sample (if consenting)	60<br><br>(+30 for DHM appointment)
	Diet Intervention group only: Instructions, meal plan and advice re diet intervention	30 minutes
**Day 2-20**	Diet Intervention Group self-administer the diet intervention as directed	
**Day 21**	**Both groups:**<br>Online: Medical history, International Physical Activity Questionnaire, Pennsylvania Insomnia Rating Scale 2, General Self-Efficacy Scale, CESDR, DASS-21, Profile of Mood States, DFS, Diet Compliance Survey, Diet Satisfaction Questionnaire (Diet Change group only)<br>C3B 503: Urine sample, Finger prick blood sample, Anthropometric measures, photospectrometry, Hopkins Verbal Learning Test, Digit Span Forwards, Matrix Reasoning<br>Douglas Hanly Moir: Serum blood sample (if consenting)	60<br><br>(+30 for DHM appointment)
	No Change group only:<br>Instructions, meal plan and advice re diet intervention provided via weblink to do at home if they desire.	
**3 month follow up**	Diet change group only:<br>Telephone follow up questions - brief survey of mood and diet over the past 3 months	5 minutes

6.2. INVESTIGATION PLAN

Method:

Prior to the first experimental day, participants will be asked to consent (Appendix 1) to the online questionnaires, then complete questions regarding: demographic details, brief medical history,

physical activity, sleep quality, general self efficacy, mood screening, and dietary habits and intake (Appendix 2). These same online questionnaires will be completed just prior to the second testing session on Day 21, with the addition of a diet satisfaction survey. Participants will then attend 2 testing sessions, 3 weeks apart (baseline & day 21). In both sessions, participants who consent to, will provide a blood sample (Douglas Hanly Moir; DHM), then attend the laboratory to provide a urine sample (in the adjacent bathroom), finger-prick-blood sample (see below), body measurements (BMI & fat mass using body composition scales), photospectrometry (a device will be held to the palm of the hand and will take a photo to measure skin colouration, which is a reliable indicator of fruit and vegetable intake) and neuropsychological tasks. Participants will be randomly assigned to either DC or NC group. During the first session only, the DC group will be instructed to increase intake of fish, fruits, vegetables, nuts, seeds, natural diary and wholegrain cereals, and decrease refined carbohydrate, sugar, fatty or processed meats and soft-drinks. Recommendations are based on the Australian Guide to Healthy Eating, as well as Mediterranean diet principles known to be associated with improved mood, incorporate BeyondBlue behavioural tips for eating well, and administered by a registered dietician via video and available to re-watch as needed. They will be provided a sample meal plan and recipes, plus a diet diary to complete during the study. Participants will be provided online access to the diet intervention video and FAQs plus a questions facility on Moodle, monitored by the research team (incl. dietician and clinical neuropsychologist). To encourage dietary adherence, participants will be texted on a weekly basis, with a dot-point reminder of the main diet goals. As an additional compliance check, they will be asked to provide grocery receipts to demonstrate purchase of the diet-intervention foods. Finally, participants are provided with a food hamper containing the main components of the recommended diet and recipes. Those in the NC will be instructed to continue their diet as usual, but on Day 21 will be offered the resources used by the DC group. Participants in the diet change group who are consenting will be contacted by telephone to complete a brief survey regarding whether they have maintained the diet change and their current mood.

Compliance will be measured using diet questionnaires (DFS and Diet Compliance Survey), and biological measures that indicate amount of intake of various nutrients (sucrose + creatinine in urine, photospectrometry which correlates with fruit and vegetable intake).

Finger prick blood sample: This will be used to measure glycosylated haemoglobin (HbA1c) as an indication of how well an individual maintains glucose homeostasis (see further information regarding collection below).

Urine and blood samples: These will be analysed for a) cytokines and kynurenine pathway metabolites as biomarkers of inflammation and b) sucrose and creatinine for biomarkers of sugar and protein intake respectively (see further information regarding collection below).

NEAF questions regarding the collection of human biospecimens:

1. What is the nature of biospecimen/s you plan to use?

Finger-prick blood, urine, serum from whole blood

2. What is the source of the biospecimen/s you wish to use?

Collected from participants recruited to this research project who are not concurrently undergoing diagnosis or treatment

2a. How and by whom will the biospecimen/s be collected?

A member of the research team will collect finger-prick blood, & urine:

The experimenter will wear gloves when obtaining the 40ul whole blood sample taken from the participant finger. The participant will wipe a test strip over their finger, across the blood sample, and then dispose of the test strip of in a biohazard waste bin once the device has (almost immediately) calculated the HbA1c value. Both experimenter and participant will immediately wipe their hands with antibacterial wipes.

For urine collection, subjects will be asked to collect their urine in a sterile 70ml specimen container. The specimen container lid will be sealed tight and placed into a biohazard bag. After providing the sample, participants clean their hands with water and soap in the sink, followed by an antibacterial wipe. The experimenter wears gloves when handling the container in biohazard bag. Urine samples are then stored in a dedicated fridge until collection.

A third party will collect serum from whole blood:

A certified and trained phlebotomist from Douglas Hanly Moir Pathology Services, Macquarie University Hospital, will be collecting the blood from the recruited subjects. About total of 10ml of whole blood will be drawn using aseptic technique and collected into 2 BD Vacutainer SST II plus plastic serum tube. The tubes will then be stored at 4 degrees until ready for collection, transport and process in a research lab to separate the serum from the tube within 3 hours in the same building (F10A). Subjects are allowed to rest for another 10-15min after collection and examine again to ensure that the subjects are not feeling anaemic from the blood collection. If such unlikely event arises, subjects will be referred to a doctor in the GP clinic for further examination in the same building.

2b. In what form will the biospecimen/s be provided to the research team?

De-identified

3. In what form will the biospecimen/s be used by the investigators in the conduct of this project?

Re-identifiable: biospecimens will be labelled using a unique idenitification code that is known only the Chief Investigators and only used to match the biospecimen to the questionnaire and neuropsychological data collected during the study.

Does the proposed research have the potential to reveal information that may be important for the health of the donor/s, their blood relatives or their community?

No. Finger-prick blood samples will be used to obtain HbA1c levels. Urine and serum blood will only be analysed for cytokine levels and kynurenine pathway upregulation. None of this information is diagnostic.

4. Will the biospecimen/s used for this project be destroyed once the project is completed?

Yes

5. Does this research involve the development of a cell line?

No

6. Provide details of the collection and management of the biospecimen/s.

Finger-prick blood samples: Using a Point-of-Care Test device, the researchers plan to take finger prick blood samples of 40ul to measure HbA1c. The 40ul whole blood sample taken from the participant finger will be disposed of appropriately once the device has (almost immediately) calculated the values for the metabolites.

Urine: Urine will be collected in a 70ml specimen container labelled with the participant's unique identification code. Participants will be asked to collect mid-stream urine and not the first part of the urine. The participant will be provided a snap lock bag to place the contained into, and this will be placed in a fridge at -4 degrees Celcius until collection by Dr Edwin Lim within 3 hours for storage at Macquarie University. Once collected, the samples will be keep on ice before aliquoting 1ml into as many tubes as possible. Samples will be stored -80 degree Celsius for later use.

Serum blood: Samples will be collected by Douglas Hanly Moir Pathology Services, Macquarie University Hospital, who will be contracted to take the samples, which will then be collected by Dr Edwin Lim for storage at Macquarie University.

Bloods will be collected using formal venepuncture (needle and syringe/vacutainer) while adhering to bio-safety and workplace-safety requirements. Samples will then be centrifuged - plasma and serum separated and aliquoted and frozen at -80 degree Celsius for later use.

Transport of urine and blood samples: Containers will be securely stored in a leak proof contained, appropriately labeled and and placed in a biohazard bag and double sealed in a secondary containment (i.e. esky) during transport.

Analysis of urine and blood samples: Researchers will wear protective clothing such as buttoned-up coats and gloves, and enclosed shoes. Centrifuge procedure will be carried out in a fitted sealed rotor, with the human biological samples contained in a leak-proof sealed tube. Samples will be kept in secured leak-proof containers with appropriate labeling, and stored in fridge or freezer. At the end of the experiment, all metal work surfaces will be disinfected with 70% ethanol and non-metal work surfaces will be cleaned with disinfectant. Samples will be chemically disinfected, and sent out for incineration as part of the contaminated waste disposal service. All consumable items will be disposed in yellow biological waste bags. These bags will be transferred to commercial contaminated waste disposal bins and disposed weekly by contracted services.

7. Describe how you will ensure that all biospecimen/s used in this project will be stored securely and describe how you will monitor this as well as the use of the biospecimen/s.

Participants will be assigned a unique identification code when they agree to participate in the study. Only the unique identification code will be used when collecting and storing the samples. No other identifying information will be noted on the samples. Information from the analysed samples will then be matched to survey and neuropsychological data using this code. All urine and blood biospecimens will be safely destroyed following publication of the data (factoring in the possibility for any peer-review suggested re-analysis of the data).

Finger-prick blood samples will be disposed of appropriately once the device has (almost immediately) calculated the HbA1c value. Urine and serum blood samples will be aliquoted and coded and stored in -80 freezers. Access to these freezers is limited to personnel working in Dr Edwin Lim's laboratory. Researchers will keep a log of samples going in and out of the freezer for analyses.

Analysis

Blood & urine sample analysis: Samples will be analysed at the Australian Proteomic Analysis Facility using a Bio-Rad 27-plex kit, allowing detection of 27 cytokines, chemokines and growth factors related to inflammation. Quantification of kynurenine pathway metabolites (KA and QA) will be performed by ultra-high performance liquid chromatography (UHPLC) and gas chromatography mass spectrometry (GC-MS), as routinely used by Dr Lim.

Statistical analysis: Normality and variance checked prior to analysis carried out in SPSS. Mood and biomarkers will be analysed using a mixed-model two-way repeated measures ANOVA with diet group (DC or NC) as the between-subjects factor and time (baseline, day 14) as the within-subjects factor. We will establish a regression model to predict the mental status (outcome) using explanatory variables such as food score, KP and inflammatory markers as predictors using logistic regression analysis. The model will be built on a stepwise manner to elucidate parameters that give the best R value. Cross interaction for potential confounders and adjustment will be applied. P values of less than 0.05 will be considered statistically significant, with Bonferroni adjustment to correct for multiple comparisons.

6.3. STUDY PROCEDURE RISKS

If a participant is distressed after completing either the DASS-21-D on screening or 3 month follow up, or the CESDR on Baseline or Day 21testing sessions, we provide a link to online emergency chat services that they can access immediately, as well as a pdf list of telephone and online resources that they can contact in an emergency or to seek more long term treatment. We expect the chance of a student reacting adversely to depression questionnaires to be very low based on previous use of this questionnaire with student cohorts. Those who do seek treatment and commence pharmacological or psychological therapy will not be automatically excluded from the study, but will need to have been on the treatment for at least 2 weeks prior to study commencement.

Blood sampling is a low risk activity but participants will be made aware that there are a number of minor complications that can result from the procedure (both at the time of blood extraction and in the participant information sheet). In order for subjects to give informed consent to blood sampling they will read and understand the following prior to blood collection:

Complications of blood sampling:

1. Syncope (fainting): This is not common in healthy volunteers but can more commonly occur if subjects are unwell or suffering from a viral infection such as a cold or flu. Subjects who are extremely apprehensive about the procedure or the sight of blood are also prone to fainting. If this does occur the subject will be laid down, and adequate ventilation with fresh air is provided. A glass of cold water often helps to alleviate the symptoms.

2. Nausea and Vomiting: Whilst a feeling of nausea is a relatively common response (especially in first time subjects), vomiting is quite uncommon in adults as a response to blood sampling.

3. Bruising and Haematoma formation: Bruising is the most common post-procedure complication. The likelihood of bruising can be greatly diminished by applying pressure to the puncture site for 5 minutes after the completion of the procedure. Haematoma formation (bleeding under the skin to form a raised swelling) can also occasionally occur and is minimised with prolonged application of pressure to the site.

4. Convulsions: Usually only seen in patients who faint. These are usually minor in nature and last less than a minute. The procedures for this are the same as for syncope, and in the unlikely event that these occur, the participant will be sent to a doctor for further examination.

6.4. PARTICIPANT RECRUITMENT AND SCREENING

Posters shall be displayed around the Macquarie University campus advertising the study, as well as online advertising (Gumtree), through social media (Facebook), and the Macquarie University Psychology Participant pool recruiting webpage and emails. Initial contact with potential participants will not be face-to-face, but over the telephone. Potential participants may call the co-investigator directly, after which they will be screened for eligibility using the criteria specified in Section 5.

6.5. PARTICIPANT ENROLMENT

Potential participants will be screened according to the inclusion/exclusion criteria previously mentioned prior to be inducted into the study. Those scoring in the elevated range on the DASS-21 Depression scale will be contacted, provided with information regarding resources to contact regarding seeking assistance, and invited to participate in the study. Participants will attend the laboratory on Day 1 and they will be randomly allocated to a test condition, and given the respective information and consent form to complete.

6.6. INFORMATION AND CONSENT

Participants will be required to sign information and consent forms prior to completing study procedures. They will be informed of the potential risks and benefits associated with participation. All participants will be told that participation is voluntary, and they may withdraw without consequence. Providing serum blood samples will be an additional consent form, and those who do not consent will not be excluded, they will simply complete all other aspects of the study.

6.7. RANDOMISATION PROCEDURE

Participants will be randomly assigned to either diet intervention or no change using an Excel generated randomization schedule. One group will receive information regarding diet intervention on Day 1, and the other will not receive that information until Day 21, after the study has finished.

6.8. END OF STUDY TREATMENT/WITHDRAWAL PROCEDURE

Participants may contact the investigators to withdraw from the study. All participants will be told that participation is voluntary, and they may withdraw without consequence.

6.9. PATIENT WITHDRAWAL

Participants may contact the investigators to withdraw from the study. All participants will be told that participation is voluntary, and they may withdraw without consequence.

7. OUTCOMES

7.1. DEFINITION OF OUTCOMES

Summary of outcome measures and schedule

Variable	Measures	Location
Self-report		
Medical history	Bioform	Online
Exercise	International Physical Activity Q'naire	Online
Sleep	Pittsburgh Insomnia Rating Scale 2	Online
Mood	CESD-R, DASS-21, POMS	Online
Diet Quality	DFS, Diet Compliance Score	Online
Self-efficacy	General Self Efficacy Scale	Online
Diet satisfaction	Diet habits and feedback survey	Online
Neuropsychological		
Memory	HVLT, Logical Memory, self-report memory Q'naire	C3B503
Intellectual Function	Matrix Reasoning	C3B503
Anthropometric		
Height	Height chart (nearest 1cm)	C3B503
Weight	Scales (nearest 0.1kg)	C3B503
Waist circumference	Tape measure (nearest 1cm)	C3B503
Biochemical		
Carotenoids	Spectrophotometry	C3B503
Triglycerides	Finger-prick blood sample	C3B503
Blood glucose level	Finger-prick blood sample	C3B503
Cytokine assay	Serum blood sample; urine	DHM/F10A; C3B b'room
Kynurenine metabolites	Serum blood sample; urine	DHM/F10A; C3B b'room
Sucrose (sugar intake)	Urine	C3B b'room

Creatinine (protein intake)	Urine	C3B b'room

8. STATISTICAL CONSIDERATIONS

1.1. SAMPLE SIZE OR POWER CALCULATION

This study will use 80 young adults (n=40 per group). Sample size was based on ability to detect relationships between the most conservative key dependent variables; diet and mood. McMillan et al (2011) showed a 10 day Mediterranean diet improved mood (d=1.34) and a recent RCT by Jacka et al. (2017) showed a 6 month diet intervention improved mood (d=1.16). There are no other whole-diet change trials to base our estimations on, therefore, we have conservatively estimated an effect size of .80, alpha level.01 (one-tailed as direction is hypothesised), power.80, a total of n = 36 participants per group is required (we aim for 40 per group based on a <10% dropout rate in the Jacka at al, 2017, study). Intention to treat analysis will be carried out for non-compliance.

1.2. PROVIDE A DETAILED ANALYSIS PLAN

Normality and variance checked prior to analysis carried out in SPSS. Non-parametric statistics will be used when assumptions for parametric methods are violated.

Mood and biomarkers will be analysed using a mixed-model two-way repeated measures ANCOVA with diet group (DC or NC) as the between-subjects factor and time (baseline, day 21) as the within-subjects factor. To assess confounding, covariates will include age, gender, Body Mass Index (BMI) physical activity levels, smoking, alcohol consumption and self-efficacy. Correlational analyses will explore dose-response effects associated with diet adherence within the Diet Intervention group. Intention to treat analysis will be employed.

We will establish a regression model to predict mood (outcome) using explanatory variables such as food score, KP and inflammatory markers as predictors using logistic regression analysis. The model will be built on a stepwise manner to elucidate parameters that give the best R value. Cross interaction for potential confounders and adjustment will be applied. P values of less than 0.05 will be considered statistically significant, with Bonferroni adjustment to correct for multiple comparisons.

9. DATA COLLECTION

9.1. PARTICIPANT REGISTRATION

N/A

9.2. FORMS AND PROCEDURE FOR COLLECTING DATA

Mood:

- CESD-R: consists of 20 feelings and behaviours that participants rate on a scale from 0 (not at all) to 4 (every day), how often they had felt that way in the past 2 weeks. This will be used as an outcome measure in the study (i.e. administered at baseline and Day 21).

- DASS-21: consists of 21 statements which participants rate on a scale from 0 (not at all) to 3 (very much so), how much it has applied to them in the past week.
- POMS: A list of 24 adjectives, which participants rate how much the word describes how they are feeling "right now".

Neuropsychological testing:

- HVLT-II: A 12 item word list is read 3 times and the participant is asked to repeat as many words as they can each time. 20-30 minutes later, participants are asked to recall the word list again.
- Logical Memory: Participants are read a short story and asked to repeat back as much as they can remember. 20-30 minutes later, participants are asked to again repeat back as much as they can remember.
- Matrix Reasoning: Participants are asked to select which option (out of 5) fits to solve a visual puzzle. There are 26 total puzzles, with participants discontinuing with incorrect response on 3 in a row.

Diet intake and habits:

Dietary Fat and Free Sugar Questionnaire (DFS): assesses frequency of consumption of 26 foods and drinks that are high in saturated fat and/or added sugar.

Diet Compliance Survey: A list of food and drink items consistent with the Mediterranean Diet Pyramid. Participants rate how often they eat them.

Diet habits survey: Series of questions regarding factors that influence diet (access to food preparation facilities, current habits etc) and which aspects of the diet intervention participants found achievable or unfeasible.

Diet satisfaction survey: Questions regarding how difficult participants found it to comply with the diet recommendations and which aspects of the diet they liked/disliked.

Bioform: comprised of demographic and medical history questions.

International Physical Activity Questionnaire: 4 questions regarding frequency and level of intensity for vigorous and moderate physical activity, walking and sitting.

Pitsburgh Insomnia Rating Scale: Two questions requiring participants to rate lack of energy due to poor sleep and sleep satisfaction.

General Self-Efficacy Scale: 8 statements that participants rate on a 5 point likert scale how much they agree.

Anthropometric measures: Participants will be asked to remove their shoes and any bulky outer layers of clothing for measurement of height, weight and waist circumference.

Biological measures:

- Finger prick blood samples: Using a Using a Point-of-Care Test device, the researchers plan to take finger prick blood samples of 40ul to measure HbA1c. The 40ul whole blood sample taken from the participant finger will be disposed of appropriately once the device has calculated the value.

- Urine sample: Participants will be asked to collect their urine in a sterile 70ml specimen container, re-identifiable using participant number. Patients will be asked to collect mid-stream urine and not the first part of the urine. The specimen container lid will be sealed tight and placed into a biohazard bag. Urine samples are then stored in the fridge until collection by Dr Edwin Lim, or a research assistant (yet to be identified – the name and details will be supplied to the ethics committee once known). Once collected, the samples will be kept on ice before aliquoting 1ml into as many tubes as possible. Samples will be stored at -80 degree Celsius for later use.

- Blood sample: A certified and trained phlebotomist from Douglas Hanly Moir, Macquarie University Hospital, will perform blood sample collection. Whole bloods will be collected using formal venepuncture (needle and syringe/vacutainer) while adhering to bio-safety and workplace-safety requirements. About total of 10ml of whole blood will be drawn using aseptic technique and collected onto 2 BD Vacutainer SST II plus plastic serum tube, de-identified participant number. The tubes will then be stored at 4 degrees until ready for collection, transport and process in a research lab to separate the serum from the tube within 3 hours. Samples will then be centrifuged - plasma and serum separated and aliquoted and frozen at -80 degree Celsius for later use.

10. QUALITY CONTROL AND ASSURANCE

10.1. CONTROL OF DATA CONSISTENCY

All tests will be administered and scored according to their respective administration manuals, ensuring consistency across sessions and participants.

10.2. AUDITS

None.

10.3. PROTOCOL AMENDMENTS

None.

11. ETHICS

11.1. INVESTIGATOR AUTHORISATION PROCEDURE

This study requires approval of ethics forms, the participant information and consent form, advertisements, and questionnaires relevant to data collection.

11.2. PATIENT PROTECTION

The responsible investigators will ensure that the study is completed in accordance with the guidelines set out in the *National Statement on Ethical Conduct in Human Research* (2007) (the *National Statement*) and the *CPMP/ICH Note for Guidance on Good Clinical Practice* and any other relevant legislation/guidelines. Any participant harm or complaints will be reported to the relevant ethics committee. Any complaint made will be treated in confidence and investigated, and participants will be informed of the outcome. Participants are free to withdraw from the study without consequence.

12. SAFETY

12.1. ADVERSE EVENT REPORTING

Any participant harm or complaints will be reported to the medical ethics committee. Any complaint made will be treated in confidence and investigated, and participants will be informed of the outcome.

12.2. SERIOUS ADVERSE EVENT REPORTING

All serious adverse events will be reported immediately to the relevant HREC. The reports will be followed by a detailed written report. Follow-up reports should identify the participant/s by unique code assigned to participants (rather than by name).

13. BLINDING AND UNBLINDING

No blinding procedures will be used in this study.

14. CONFIDENTIALITY AND STORAGE AND ARCHIVING OF STUDY

All data will be stored securely. Paper copies will be kept in secure filing cabinets; online data will be stored on password protected computers. All data will be kept for up to 5 years after publication as per standard for publication in Psychology journals.

Biological samples: identification, safe storage and monitoring of the biospecimens

Participants will be assigned a unique identification code when they agree to participate in the study. Only the unique identification code will be used when collecting and storing the samples. No other identifying information will be noted on the samples. Information from the analysed samples will then be matched to survey and neuropsychological data using this code.

All samples will be aliquoted and coded and stored in -80 freezers. Access to these freezers is limited to personnel working in the lab. Researchers will keep a log of samples going in and out of the freezer for analyses.

15. TRIAL SPONSORSHIP AND FINANCING

This research is supported by a Society for Mental Health Research Early Career Researcher Project Grant.

17. REFERENCES

Hendrie, G., Baird, D., Golley, S., Noakes, M. (2016). CSIRO Healthy Diet Score 2016.

Kiecolt-Glaser, J. K. 2010. Stress, Food, and Inflammation: Psychoneuroimmunology and Nutrition at the Cutting Edge. *Psychosom Med 72:* 365-369.

Hure, A., McEvoy, M. & Attia, J. (2014). Associations of depression with C-reactive protein, IL-1, and IL-6: a meta-analysis. *Psychosomatic Medicine 71,* 171–186.

Lai, J., HIles, S., Bisquera, A., Howren, M.B., Lamkin, D.M., Suls,J. 2009.A systematic review and meta-analysis of dietary patterns and depression in community dwelling adults. *Am J Clin Nutr, 99,* 181-197.

Jacka, F.N., ET AL. (2017). A randomised controlled trial of dietary improvement for adults with major depression (the 'SMILES' trial). *BMC Medicine, 15,* 1-13.

McMillan, L., Owen, L., Kras, M. & Scholey, A. 2011. Behavioural effects of a 10-day Mediterranean diet. Results from a pilot study evaluating mood and cognitive performance. *Appetite, 56;* 143-147.

Myint, A.M., Kim, Y.K., Verkerk, R., Scharpe, S., Steinbush, H. & Leonard, B. 2007. Kynurenine pathway in major depression: Evidence of impaired neuroprotection. *J Affective Disord 98:* 143-151.

Orygen, The National Centre of Excellence in Youth Mental Health, Research Bulletin Issue 03.

18. APPENDICES

Appendix 1: Society for Mental Health Research Early Career Research grant application

Appendix 2: Participant information and consent forms

Appendix 3: Copy of questionnaires

Appendix 4: Diet intervention script and materials

Appendic 5: Telephone follow up questions

Appendix 6: Recruitment materials

Appendix 7: Mental Health Resources
